# The Effects of Infliximab on Laminin, NF*κ*B, and Anti-TNF Expression through Its Effect on Ischemic Liver Tissue

**DOI:** 10.1155/2016/1738430

**Published:** 2016-04-07

**Authors:** Remzi Adnan Akdogan, Yildiray Kalkan, Levent Tümkaya, Halil Rakici, Elif Akdogan

**Affiliations:** ^1^School of Medicine, Department of Gastroenterology, Recep Tayyip Erdoğan University, 53100 Rize, Turkey; ^2^School of Medicine, Department of Embriology and Histology, Recep Tayyip Erdoğan University, 53100 Rize, Turkey; ^3^School of Medicine, Department of Hematology, Recep Tayyip Erdoğan University, 53100 Rize, Turkey

## Abstract

The aim of this study was to investigate the possible protective effects of infliximab on expression of laminin, anti-TNF, and NF*κ*B in the rat hepatic cells after ischemia/reperfusion (I/R). A total of 30 male Wistar albino rats were divided into three groups: Control (C), sham I/R (ISC), and I/R+ infliximab (ISC inf); each group comprised 10 animals. C group animals underwent laparotomy without I/R injury. In ISC groups after undergoing laparotomy, 1 hour of superior mesenteric artery ligation was done, which was followed by 1 hour of reperfusion. In the ISC inf group, 3 days before I/R, infliximab (3 mg/kg) was administered intravenously. All animals were killed at the end of reperfusion and hepatic tissue samples were obtained for histopathological and histochemical investigations in all groups. Laminin, anti-TNF, and NF*κ*B immunoreactivity were performed for all groups. ISC caused severe histopathological injury including mucosal erosions, inflammatory cell infiltration, necrosis, hemorrhage, and villous congestion. Infliximab treatment significantly attenuated the severity of intestinal I/R injury and it is shown by laminin, anti-TNF, and NF*κ*B immunoreactivity. Because of its anti-inflammatory and antioxidant effects, infliximab pretreatment may have protective effects on hepatic cells in the experimental intestinal I/R model of rats.

## 1. Introduction

Ischemia and reperfusion injury of the liver are still an important clinical problem during shock, organ transplantation, and surgery. Clinical liver cells are affected and cell damage develops particularly following acute hypoxia and hypotension and it can lead to liver failure. Liver function is expected to be corrected with clinical improvement. The oxygen-free radicals produced by the activated Kupffer cells play an important role in the formation of the resultant ischemic damage in liver. Proinflammatory mediators such as TNF-alpha, NF*κ*B (nuclear factor kappa-light-chain-enhancer of activated B cells), and interleukins (IL-1, IL-8) are the main factors related to cell damage. TNF-alpha is one of the major inflammatory mediators in liver and the other organs. While healing after ischemia and reperfusion injury, laminin is one of the major proteins of the extracellular matrix basement membrane. It has an important role in tissue healing. Infliximab is a chimeric human-murine monoclonal antibody against TNF-alpha. Anti-TNF-*α* agents are biological compounds which induce remission by reducing inflammation and providing tissue healing effect especially in inflammatory bowel diseases. Also, it is reported that inhibition or neutralization of TNF-alpha decreases neutrophil infiltration in the liver and it reduces I/R injury [1]. Therefore, inhibition or neutralization of TNF-alpha may be helpful in the treatment of ischemic conditions of different organs. In this study, we planned to investigate the effects of infliximab on laminin, NF*κ*B, and anti-TNF expression through its effect on ischemic liver tissue. Therefore, the determination of infliximab effect, drug-tissue interactivity, the positive or negative nature of the drug effect, and their usage as a scientific data is important in terms of drug side effects and overdose. It is expected to provide a significant contribution over animal and human health and their welfare. There is no indicated drug therapy for ischemic liver injuries other than support treatment. The role of infliximab in the improvement of liver injury due to its anti-TNF properties will reveal the role of the antilaminin, NF*κ*B, and anti-TNF expression with its possible effects. The following results may lead to possible new treatment options.

## 2. Materials and Methods

In our study, a total of thirty (ten in each group), 7-8 months old and 250–350 grams weighted Sprague-Dawley male rats were used. All animals were fed with 7-8 mm pellets rat chow and water ad libitum. Automatic photoperiod with white fluorescein was used to provide 12 h light and dark cycles (06.00–18.00) and temperature was determined as 21 ± 3°C and humidity rate as 55–60%. The methods used for animal experiments were organized according to the National Institute of Health Guide for the Care and Use of Laboratory Animals protocol. The necessary permits were obtained from Rize University Faculty of Medicine Animal Experiments Ethics Committee (Date: 25/11/2011, Decision number 26).

### 2.1. Methods

The animals were divided into 3 groups consisting of 10 subjects in each group. Group 1 (C) was named as the sham-control group. In Group 2 (ISC), Pringle maneuver was performed to obtain the model of hepatic ischemia. Group 3 (ISC inf) was started on infliximab 3 mg/kg/day three days prior to the application of ischemia. No drug was administered to sham group and the abdominal region was opened and closed with surgery without the application of ischemia model. All the animals were decapitated at the end of the experiment under deep anesthesia with sodium pentothal (50–60 mg/kg) and liver tissues were obtained for histopathological and immunohistochemical examination.

After being labeled, the liver was left at a 10% neutral formaldehyde solution. Following twenty-four hours of waiting in the fixative, it was washed under water for 4–6 hours and embedded in liquid paraffin after passing automated tissue processing from ethanol-xylene series (Citadel 2000 Thermo Fisher Scientific Shandon, England). The tissue was cut 4–6 *μ*m for H&E stain and 3-4 *μ*m for immunohistochemistry staining. The appropriate parts detected under a light microscope were examined at different magnifications and photographed.

The sections obtained for immunohistochemical staining were left for 10 minutes in xylene twice and after passing in alcohol series (70–99%) for 5 minutes were kept for 10 minutes in 3% H_2_O_2_ solution. After washing with PBS, they were heated four times in citrate buffer for 5–10 minutes under 800 Watt power and allowed to stand for 20 minutes in secondary blocking agent. Each preparation was allowed to stand 60–75 minutes in various dilutions (1/200–1/250) of the primary antibody of antilaminin (CoD: ab11575, Abcam plc, Cambridge CB4 0FL, UK), anti-NF*κ*B p65 (CoD: ab7970 Abcam plc, Cambridge CB4 0FL, UK), and anti-TNF-*α* (CoD: ab1793, Abcam plc, Cambridge CB4 0FL, UK). The Diaminobenzidine (DAB) solution was used as chromogenic and stained with Mayers hematoxylin for contrast dye. PBS, instead of primary antibodies, was used for negative controls. Preparations were closed off with suitable agents and photographed. According to the blindly scoring evaluations performed by two histologists, the positivity was divided into the following four categories according to the % value: mild (+), moderate (++), severe (+++), and very severe (++++).

### 2.2. Analysis and Results

The sections obtained by immunohistochemical staining were evaluated and graded by two blind histologists and the positivity were expressed in terms of percentage and divided into four grade categories as follows: mild (+), moderate (++), severe (+++), and very severe (++++). The obtained values were statistically compared with each other.

## 3. Results

**Figure 1:**
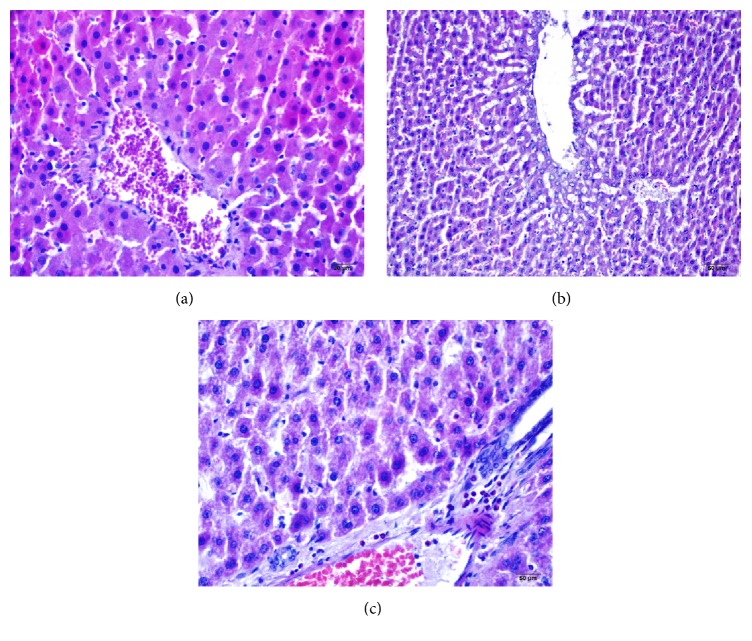
(a) Control group, (b) ISC group, and (c) ISC inf group: sections stained with hematoxylin-eosin.

In the histological examination performed by hematoxylin and eosin staining in the control groups, any histopathological changes were not encountered and the artery and the vein and eluent channel trio in the portal areas of the liver and connective tissue cells were found to have normal morphological appearance. Hepatocytes with basophilic nucleus and eosinophilic cytoplasm core forming polyhedral structures were observed. Sinusoids and hepatic cord structures were observed regularly with flat basophilic Kupffer cells core located in the intermediate region (Table 1, Figure 1).

In the ISC group, fragmentation with severe focal necrosis, hemorrhage, and increases in infiltration of neutrophil granulocytes were observed in the hepatic cord. Dilation and few neutrophil granulocytes with round nuclei and nuclei divided into segments were detected in the sinusoidal area. In the examination of hepatocytes, intensive apoptotic bodies were determined. Besides apoptotic bodies present in the apoptotic cells, curling core, protruding edges of chromatin (chromatin margination), and basophilic concentrations were also observed. Intense edema and dilation were found in the central vein structure. Cell degeneration was observed predominantly in area close to the central vein and in the periphery it was observed that degeneration was reduced and cell clusters with normal histological appearance were also observed. Heavy loss due to swelling of the epithelial bile duct was found.

In the ISC inf groups, disintegration and dilatation of hepatitis cord were less observed and also an increase in the reduction of necrotic structures and hemorrhagic areas was present. Compared to the ISC group, a significant reduction particularly in the number of neutrophils and eosinophil cell infiltration in the portal area were detected. A significant reduction in the dilatation of hepatic cords compared to the ISC group was determined. While degeneration was detected in the hepatocyte cells near the portal area, in the peripheral areas of the portal healthy appearance, hepatocytes were more present. The number of apoptotic cells was found lower compared to the ISC group and higher when compared to the control group. We examined the decreasing amount of cell loss related to swelling of the biliary duct epithelial cells, and beside these findings we also observed the vacuolization of biliary duct epithelial cells.

Several positive Kupffer cells and oval basic nuclear structure of these cells following staining with TNF-alpha and NF*κ*B-p65 were determined in both ISC and ISC inf groups. In addition, condensed chromatin in the nucleus was also detected. Mononuclear cells were observed to be dispersed in the periportal area. Immunoreactive degree of these cells was observed as (++) (35%).

Severe immunopositivity caused by increase of laminin (+++) was found in ischemia and reperfusion model obtained by the Pringle maneuver as a result of both histopathologic and immunohistochemical examination.

**Figure 2:**
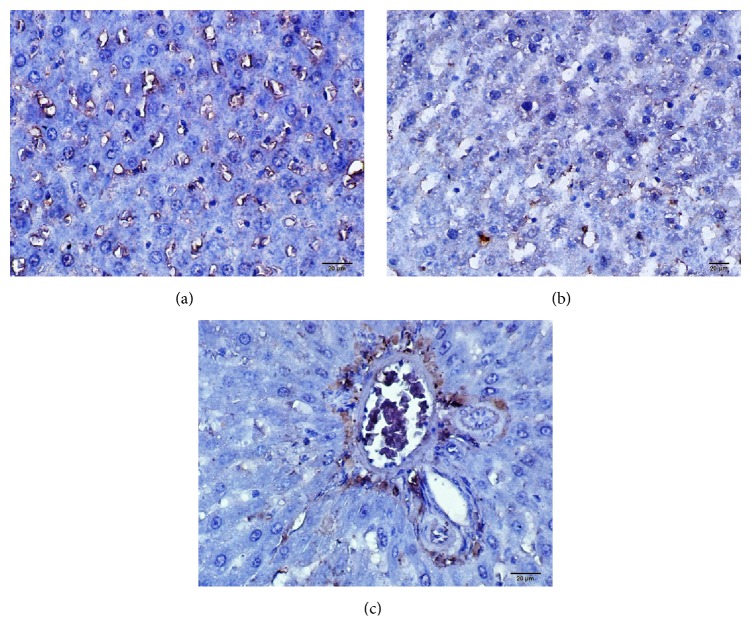
(a) Control group, (b) ISC group, and (c) ISC inf group: immunohistochemical staining of antilaminin.

Increase in the amount of laminin as a fibrosis indicator was demonstrated by means of immunopositivity reaction. Immunoreactivity in fibrotic portal areas was detected strongly. The areas with the strongest immunoreactivity (+++) were the perisinusoidal and fibrotic septa regions. In the evaluation of all the rats in the control group, the level of laminin was found to be intense in the portal area and central vein wall. Immunoreactivity rate for laminin along the sinusoidal walls was found to be (++) (45%). Of the application groups, this rate was found to be (+++) (62%) in the ISC group and (++) (65%) in the ISC inf group. The accumulation of laminin in the walls of bile duct in the portal area was defined as intense immunoreactivity (+++) (Table 2, Figure 2).

**Figure 3:**
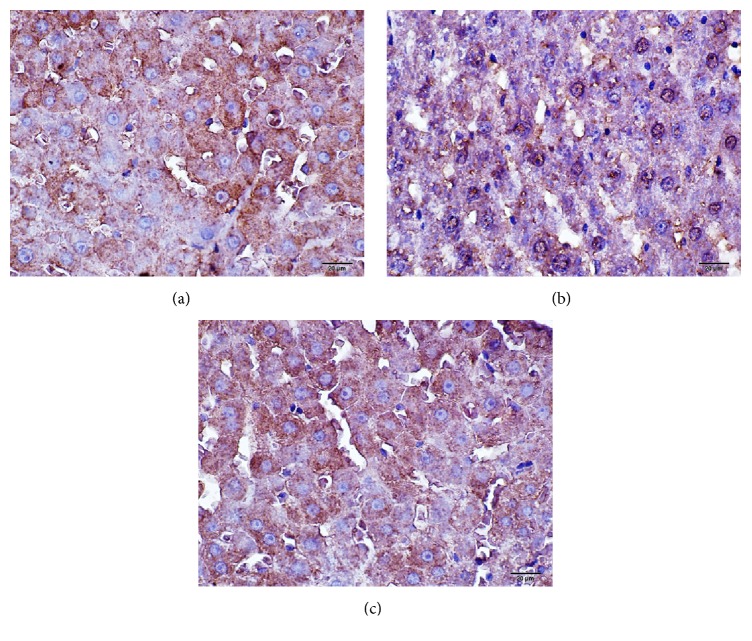
(a) Control group, (b) ISC group, and (c) ISC inf group: anti-NF*κ*B p65 immunopositivity shown in the liver tissues by the method of immunoperoxidase in immunohistochemical staining.

Anti-NF*κ*B p65 immunopositivity in liver tissue shown in immunohistochemical staining by the immunoperoxidase method was found to be (++) 10%, (+++) 10%, and (++++) 80% in Group C, (+) 20%, (++) 65%, and (+++) 15% in the ISC group, and (++) 5%, (+++) 20%, and (++++) 75 % in the ISC inf group (Table 2, Figure 3).

**Figure 4:**
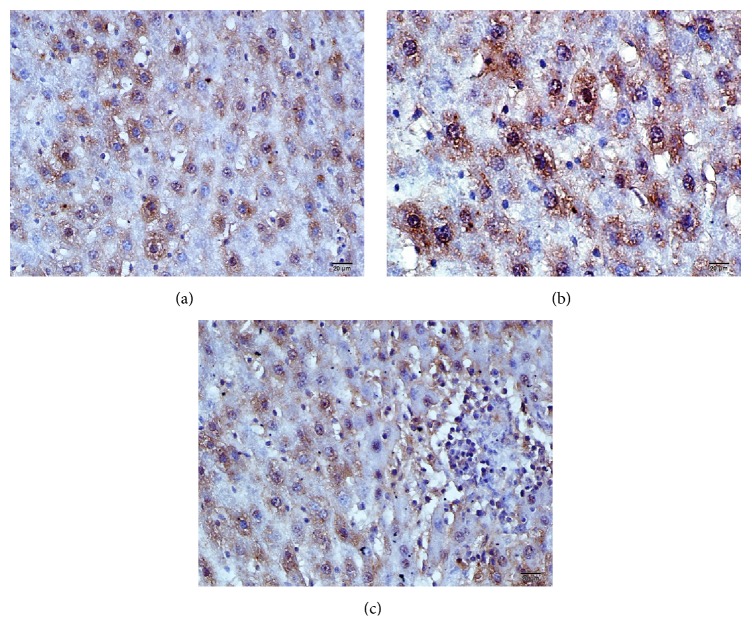
(a) Control group, (b) ISC group, and (c) ISC inf group: anti-TNF-*α* immunopositivity shown in the liver tissues by the method of immunoperoxidase in immunohistochemical staining.

Anti-TNF-*α* immunopositivity in liver tissue shown in immunohistochemical staining by the immunoperoxidase method was found to be (++) %70, (+++) %10, and (++++) %20 in Group C, (++) %15, (+++) %20, and (++++) %65 in the ISC group, (++) %5, (+++) %20, and (++++) %75 in the ISC inf group (Table 2, Figure 4).

## 4. Discussion

Ischemia and reperfusion injury in the liver are still an important issue during shock, transplantation, and surgery. Liver cells are highly sensitive for ischemia and reperfusion and cell damage develops rapidly following acute hypoxia and hypotension. Oxygen-free radicals play an important role in the development of ischemic damage. Proinflammatory mediators such as TNF-alpha, NF*κ*B, and interleukins are the main factors for cell injury [2]. Laminin is one of the major proteins of the extracellular matrix basement membrane which has an important role in tissue healing. The expression of laminin in the liver used in our study as indicator of fibrosis is found to be severely immunopositive (+++) from the histopathologic and immunohistochemical analysis, in the ischemic reperfusion model of the liver obtained by the Pringle maneuver. This rate was found to be (+++) (62%) in the ISC group and (++) (65%) in the ISC inf group. It was determined that infliximab causes a decrease in the expression of laminin.

TNF-alpha gene transcription is regulated principally by NF*κ*B. NF*κ*B is a nuclear transcription factor found in the cytoplasm of endothelial cells, Kupffer cells, and hepatocytes, and it is associated with the immune response and inflammation. NF*κ*B plays an important role in the development of acute cell damage in both hot and cold ischemia [3, 4]. Mahmoud et al. reported that inhibition of TNF-*α* protects against hepatic ischemia-reperfusion injury in rats via NF*κ*B dependent pathway [5]. NF*κ*B p65 expression in the Kupffer cells stimulates inflammation through cytokines and protects the cell. In our study, severe NF*κ*B p65 immunopositivity was detected in endothelial cells, Kupffer cells, and hepatocytes. Anti-NF*κ*B p65 immunopositivity in liver tissue shown in immunohistochemical staining by the immunoperoxidase method was found to be similar between ISC inf and C group and it was thought that infliximab carries protective effect on cells over reperfusion after ischemia.

Infliximab is a chimeric human-murine monoclonal antibody against TNF-alpha. Anti-TNF-*α* agents are biological compounds which induce remission by reducing inflammation and providing tissue healing effect especially in inflammatory bowel diseases. The protective effects of infliximab in ischemia-reperfusion injury especially in the small intestine and lungs have been shown in several studies [6, 7]. Pech et al. showed that perioperative infliximab application has protective effects on ischemia-reperfusion in experimental small bowel transplantation in rats [8]. The main sources of TNF-alpha in the liver are the Kupffer cells. In our study, TNF-alpha immunoreactivity was detected to be extremely positive in Kupffer cells. Anti-TNF immunopositivity was detected to be similarly positive in both ISC and C groups. These findings suggest TNF-alpha levels are increased in the liver tissue secondary to ischemia and it is the right target for the infliximab, a molecule used as a therapeutic agent for inflammatory diseases.

The protective effect of infliximab on ischemia and reperfusion injury of the liver has been demonstrated through the expression of laminin, anti-TNF, and NF*κ*B. Further study providing information regarding the effective dose range and possible side effects will make the use of this agent as therapeutic and/or preventive drug for this indication in humans possible.

## Figures and Tables

**Table 1:** Histopathological examination of liver tissue.

Group	Hepatocytes degeneration Median ± SD	Central canal dilatation Median ± STD	Increase of connective tissue and edema in the portal area Median ± SD	Bile duct epithelial degeneration Median ± SD	Sinusoidal dilation Median ± SD
C	0.00 ± 0.42	1.00 ± 0.42	0.00 ± 0.48	0.00 ± 0.32	0.00 ± 0.42
ISC	3.00 ± 0.47^a^	4.00 ± 0.42^a^	3.00 ± 0.32^a^	2.00 ± 0.32	3.00 ± 0.42^a^
ISC inf	2.00 ± 0.67^b,c^	3.00 ± 0.67^b,c^	2.00 ± 0.42^b,c^	1.00 ± 0.57^b,c^	2.00 ± 0.00^b,c^

^a^In the statistical evaluations performed, significant differences were observed between C and ISC groups in terms of hepatocyte degeneration, central canal dilatation, increase of connective tissue and edema in the portal area, bile duct epithelial degeneration, and sinusoidal dilation according to the Kruskal-Wallis test (*p* < 0.05).

^b^In the statistical evaluations performed, significant differences were observed between C and ISC inf groups in terms of hepatocyte degeneration, central canal dilatation, increase of connective tissue and edema in the portal area, bile duct epithelial degeneration, and sinusoidal dilation according to the Kruskal-Wallis test (*p* < 0.05).

^c^In the statistical evaluations performed, significant differences were observed between ISC and ISC inf groups in terms of hepatocyte degeneration, central canal dilatation, increase of connective tissue and edema in the portal area, bile duct epithelial degeneration, and sinusoidal dilation according to the Kruskal-Wallis test (*p* < 0.05).

**Table 2:** Antilaminin, anti-NF*κ*B p65, and anti-TNF-*α* immunopositivity shown in the liver tissues by the method of immunoperoxidase in immunohistochemical staining.

Group	Antilaminin	Anti-NF*κ*B p65	Anti-TNF-*α*
C	3.00 ± 0.47	4.00 ± 0.42	2.00 ± 0.32
ISC	1.00 ± 0.67^a^	2.00 ± 0.32^a^	4.00 ± 0.48
ISC inf	2.00 ± 0.42^b,c^	4.00 ± 1.05^b,c^	3.00 ± 0.47^b,c^

^a^In the statistical evaluations performed, significant differences were observed between C and ISC groups in terms of antilaminin, anti-NF*κ*B p65, and anti-TNF-*α* immunopositivity according to the Kruskal-Wallis test (*p* < 0.05).

^b^In the statistical evaluations performed, significant differences were observed between C and ISC inf groups in terms of antilaminin, anti-NF*κ*B p65, and anti-TNF-*α* immunopositivity according to the Kruskal-Wallis test (*p* < 0.05).

^c^In the statistical evaluations performed, significant differences were observed between ISC and ISC inf groups in terms of antilaminin, anti-NF*κ*B p65, and anti-TNF-*α* immunopositivity according to the Kruskal-Wallis test (*p* < 0.05).

## References

[1] Colletti L. M., Remick D. G., Burtch G. D., Kunkel S. L., Strieter R. M., Campbell D. A. (1990). Role of tumor necrosis factor-alpha in the pathophysiologic alterations after hepatic ischemia/reperfusion injury in the rat. *The Journal of Clinical Investigation*.

[2] Teoh N. C. (2011). Hepatic ischemia reperfusion injury: contemporary perspectives on pathogenic mechanisms and basis for hepatoprotection-the good, bad and deadly. *Journal of Gastroenterology and Hepatology*.

[3] Takahashi Y., Ganster R. W., Ishikawa T. (2001). Protective role of NF-*κ*B in liver cold ischemia/reperfusion injury: effects of I*κ*B gene therapy. *Transplantation Proceedings*.

[4] Kuboki S., Okaya T., Schuster R. (2007). Hepatocyte NF-*κ*B activation is hepatoprotective during ischemia-reperfusion injury and is augmented by ischemic hypothermia. *American Journal of Physiology-Gastrointestinal and Liver Physiology*.

[5] Mahmoud M. F., El Shazly S. M., Barakat W. (2012). Inhibition of TNF-*α* protects against hepatic ischemia–reperfusion injury in rats via NF-*κ*B dependent pathway. *Naunyn-Schmiedeberg's Archives of Pharmacology*.

[6] Pergel A., Kanter M., Yucel A. F., Aydin I., Erboga M., Guzel A. (2012). Anti-inflammatory and antioxidant effects of infliximab in a rat model of intestinal ischemia/reperfusion injury. *Toxicology and Industrial Health*.

[7] Guzel A., Kanter M., Guzel A., Pergel A., Erboga M. (2012). Anti-inflammatory and antioxidant effects of infliximab on acute lung injury in a rat model of intestinal ischemia/reperfusion. *Journal of Molecular Histology*.

[8] Pech T., Fujishiro J., Finger T. (2012). Perioperative infliximab application has marginal effects on ischemia-reperfusion injury in experimental small bowel transplantation in rats. *Langenbeck's Archives of Surgery*.

